# The association of *CDKN2BAS* gene polymorphisms and intracranial aneurysm: A meta-analysis

**DOI:** 10.1097/MD.0000000000023209

**Published:** 2020-12-04

**Authors:** Ting Yu, Hailong Jiang, Yunren Fan, Yunfeng Xu, Ning Wang

**Affiliations:** a Department of Neurosurgery, Tiantai People's Hospital, Taizhou; b Department of Neurosurgery, Zhuji People's Hospital, Zhuji City, Zhejiang Province, PR China.

**Keywords:** Intracranial aneurysm, subarachnoid hemorrhage, CDKN2BAS

## Abstract

Intracranial aneurysm (IA) is one of the main causes of subarachnoid hemorrhage (SAH) leading to a high percentage of disability and mortality worldwide. In addition to environmental factors, the risk of rupture or prognosis of intracranial aneurysm is also closely related to gene. Thus, a lot of genetic studies have been used to explore associated risk genes as well as variant loci of intracranial aneurysm and found several chromosome variates including 9p21.3 (CDKN2BAS) related to Intracranial aneurysm. However, due to differences in population and the existence of SNP, it is still not determined that whether these genetic changes can be identified as independent risk factors for intracranial aneurysm. Therefore, we performed a meta-analysis of CDKN2BAS SNPs to explore its association with intracranial aneurysms and the results show a significance relation between rs10757272, rs1333040, and rs6475606 with intracranial aneurysm. This will open a new perspective for future intracranial aneurysm gene research and therapy.

## 1 Introduction

Intracranial aneurysm (IA) are balloon-like dilations of the intracranial arterial wall that usually located at the branching points of the major arteries at the base of the brain.^[1,2]^ Intracranial aneurysm is one of the main causes of subarachnoid hemorrhage, which leads to a high percentage of disability and mortality after SAH worldwide.^[3]^ In addition to environmental factors including smoking, drinking, hypertension, diabetes, and so on, gene is also related to intracranial aneurysm. Since the family history of Intracranial aneurysm, genetics is considered as a major risk factor.^[4]^ The prevalence of first-degree relatives of SAH patients is 2 to 6 times higher than that of the control group.^[5–8]^ Moreover, it also seems that a family history may be a crucial factor influencing the risk of aneurysm rupture or the prognosis of intracranial aneurysm. Thus, plenty of genetic studies have been performed to identify associated risk genes and loci of intracranial aneurysm during these years.

Genome-wide association study (GWAS) is a critical approach in genetic research. Several genes have been found to be closely related to intracranial aneurysms by using GWAS, including chromosome 4q31.23 (EDNRA), 8q12.1 (SOX17), 9p21.3 (CDKN2A/CDKN2B/CDKN2BAS), 10q24.32 (CNNM2), 12q22, 13q13.1 (KL/STARD13), 18q11.2 (RBBP8), and 20p12.1.3.^[9–12]^

However, whether some of these chromosomal genes can be identified as true risk factors for intracranial arteries remains to be discussed due to the influence of population stratification and confounding nongenetic factors.^[13]^ The effect of genetic variants associated with intracranial aneurysm varies among different populations because of the different linkage disequilibrium (LD) structures across populations and potential interaction between genetic variants and environment factors.

CDKN2BAS located in chromosome 9p21, and it is associated with a variety of human diseases, including glioma, prostate cancer, stomach cancer, pancreatic cancer, leukemia, colorectal cancer, lung cancer, diabetes, and aneurysm.^[2,3]^ Recently, the studies of GWAS in Asian and Caucasian population identified several CDKN2BAS single-nucleotide polymorphisms (SNPs) associated with intracranial aneurysm. Hence, a meta-analysis was designed to explore the relationship between CDKN2BAS SNPs and intracranial aneurysms.

## 2 Methods

The meta-analysis was performed based on *Preferred Reporting Items for Systematic Reviews and Meta-Analyses* (PRISMA) guidelines.^[14]^ Since this study is a meta-analysis, ethical approval is not required.

### 2.1 Data sources and searches methods

We searched the following databases: PubMed, Chinese Biomedical (CBM), web of science, and EMBASE (from inception through March 2019). The following search terms were performed to identify the studies which investigate the association of CDKN2BAS SNP and intracranial aneurysm: ([polymorphisms OR variant OR variation OR genotype] AND [CDKN2BAS or Anril]) AND aneurysms. In case of the omissions of the potential studies, additional studies were identified via searching the reference lists of included studies and review articles.

### 2.2 Eligibility criteria and study selection

Inclusion criteria:

1. Studies investigating the association of the CDKN2BAS polymorphisms with intracranial aneurysm risk;
2. The odds ratio (OR) or relative risk (RR) with 95% confidence interval (CI) could be acquired or calculated from included studies.
3. Patients in eligible studies diagnosed by computed tomography angiography (CTA), magnetic resonance angiography (MRA) or digital subtraction angiography (DSA) has at least one intracerebral aneurysm.

### 2.3 Exclusion criteria

1. Animal studies.
2. Case report.
3. Review articles.
4. Studies investigating a population with an underlying connective tissue disorder or other genetic disorders associated with intracranial aneurysm.

### 2.4 Data extraction

Three investigators (TY, HLJ, and YRF) independently screened the titles and abstracts of the texts. Then, the full-text was further assessed. YFX independently extracted data from included studies and supplementary materials. The data including: Author name, Year of publication, Sample size (case/control), Mean age, Race, Gender, OR with 95% CI, SNPs, Genotype distribution in cases and controls. Inconsistencies were discussed and resolved by senior researches (NW).

### 2.5 Statistical analysis

The ORs and 95% CIs under allelic comparison were calculated, respectively. The strengths of the associations between CDKN2BAS polymorphisms and intracranial aneurysm were estimated via the above-mentioned ORs with 95% CI. All data were analyzed by using the STATA 12, a random-effects model (DerSimonian–Laird) was used to calculate the pooled ORs because of the possible heterogeneity. The *I*^2^ statistic was calculated to assess the degree of heterogeneity among the studies, significant heterogeneity was defined as *P* < .05 and *I*^2^ > 50%.^[15]^ Subgroup analysis was used to further identify the factors influencing heterogeneity. Publication bias were evaluated through Begg's funnel plot analysis and Egger's tests (significant: *P* < .1).

## 3 Results

### 3.1 Study selection

**Figure 1:**
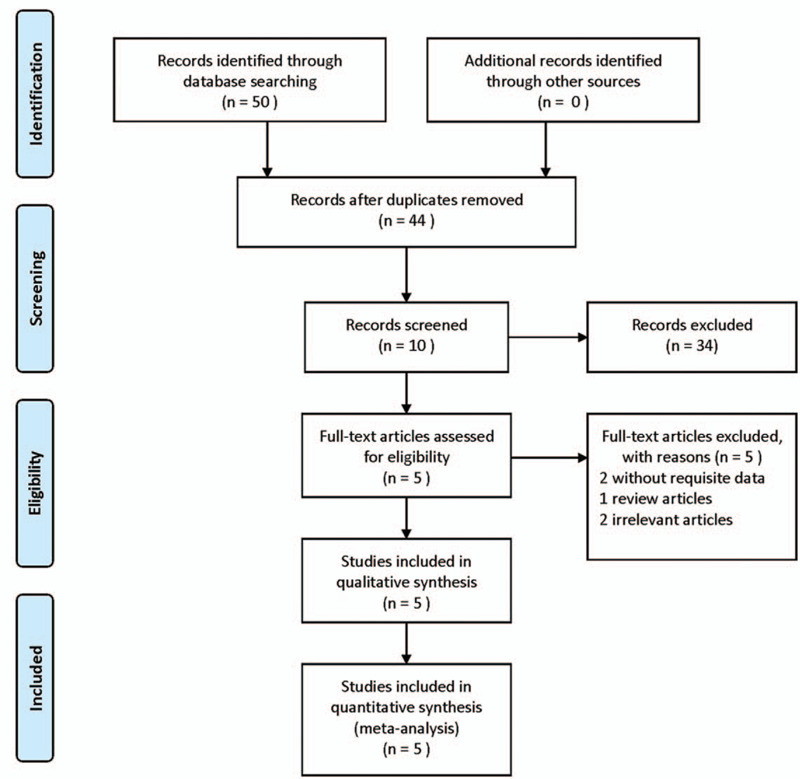
Flow chart demonstrating search strategy for meta-analysis.

As shown in Figure 1, a total of 50 articles were gathered from PubMed (12 articles), web of science (31 articles), Embase (7 articles), and CBM (0 articles). First, 6 duplicated articles were removed. After screening the titles and abstracts, 34 articles were excluded, 10 articles were undergone further assessment. After a full-text assessment, the main reasons for exclusion were: 2 articles without needed data, 1 review, 2 articles studied irrelevant SNPs. Eventually, 5 articles including 8 studies were included in the present meta-analysis: rs10757272-two studies,^[2,3]^ rs1333040-three studies,^[2,3,12]^ rs6475606-two studies,^[9]^ and rs10733376-one study.^[3,16]^

### 3.2 Study characteristics

The detailed data of the included study characteristics were shown in Table 1. The combined population size of 8 studies totaled 24,685 patients (intracranial aneurysm: 7150, controls: 17,535). These eligible patients were identified via inspection methods including CTA, MRA, DSA, or surgery. Two studies^[9,16]^ investigated the association between CDKN2BAS polymorphisms and intracranial aneurysm in Caucasian population, others investigated in Asian population. The genotype data and the OR values under allelic comparison (TT vs TC & CC) from each clinical study were shown in^[16]^Table 2. The results showed the frequencies of TT, CT, and CC genotype in both case and control group, and then the OR values were calculated under allelic comparison. Notably, four include studies directly displayed the ORs with 95% CI instead of the data.

**Table 1:** Characteristics of studies included in the meta-analysis.

Study	Year	CDKN2BAS SNPs	Sample size (case/control)	Age (mean ± SD) (case/control)	Race	Gender (female/male) (%)
Yunchang Chen	2017	rs10757272 (TC)	200/200	52.69 ± 11.5/49.99 ± 13	Asian	61.5
Yunchang Chen	2017	rs1333040 (TC)	200/200	52.69 ± 11.5/49.99 ± 13	Asian	61.5
Yunchang Chen	2017	rs6475606 (TC)	200/200	52.69 ± 11.5/49.99 ± 13	Asian	61.5
Siew-Kee Low	2012	rs10757272 (TC)	2431/12,696	60.1 ± 11.1/56.9 ± 13.7	Asian	>50
Siew-Kee Low	2012	rs1333040 (TC)	2431/12,696	60.1 ± 11.1/56.9 ± 13.7	Asian	>50
Tatiana Foround	2012	rs6475606 (TC)	1483/1683	53.9 ± 12.2/53.9 ± 9	Caucasian	>50
Tatiana Foround	2014	rs10733376 (TC)	2617/2548	53.9 ± 12.2/53.9 ± 9	Caucasian	>50
Hirokuni Hashikata	2010	rs1333040 (TC)	419/408	60.5 ± 13.6/62 ± 10.1	Asian	66.1

**Table 2:** ORs for the association between different polymorphisms and intracranial aneurysm.

Study	Year	CDKN2BAS SNPs	Case		Control		OR	LCI	UCI
			TT	TC/CC	TT	TC/CC			
Yunchang Chen	2017	rs10757272 (TC)	82	97/21	88	90/22	1.131	0.76	1.681
Yunchang Chen	2017	rs1333040 (TC)	116	65/19	90	92/18	1.722	1.159	2.558
Yunchang Chen	2017	rs6475606 (TC)	118	63/19	88	95/17	1.831	1.232	2.723
Siew-Kee Low	2012	rs10757272 (TC)	1155	1034/234	5370	5727/1594	1.21	1.13	1.3
Siew-Kee Low	2012	rs1333040 (TC)	–	–	–	–	1.16	1.09	1.25
Tatiana Foround	2012	rs6475606 (TC)	–	–	–	–	1.36	1.22	1.52
Tatiana Foround	2014	rs10733376 (TC)	–	–	–	–	1.34	1.23	1.45
Hirokuni Hashikata	2010	rs1333040 (TC)	203	180/36	170	187/51	1.28	1.04	1.57

### 3.3 Data synthesis

**Figure 2:**
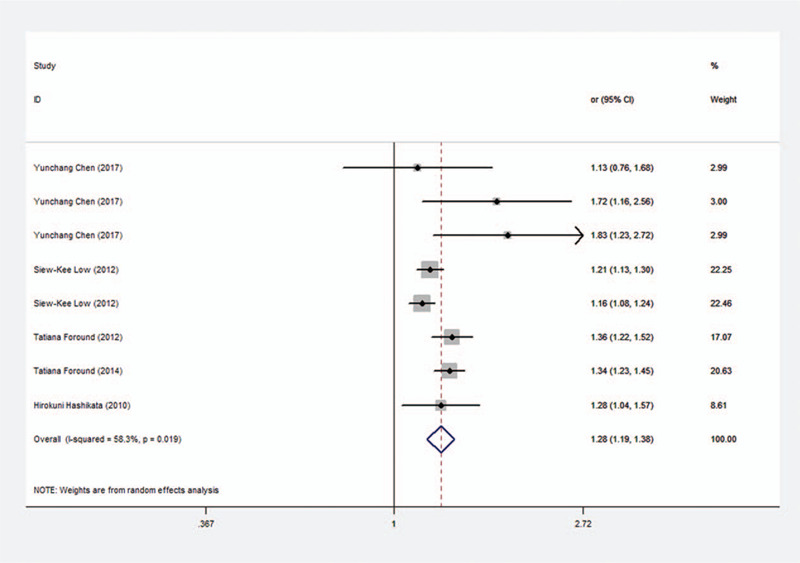
Forest plot of the association between intracranial aneurysm and the CDKN2BAS SNPs (rs10757272 or its proxy) under per-allele comparison.

In light of previous studies, the frequency of T allele is strongly associated with the risk of intracranial aneurysm among the SNPs of CDKN2BAS, including rs10757272, rs1333040, rs6475606, and rs10733376. Hence, allelic comparison (TT/TC + CC) was used to detect the correlation between T allele and intracranial aneurysm risks. The results indicated that the CDKN2BAS SNPs were significantly associated with intracranial aneurysm **(**Fig. 2, OR, 1.281; 95% CI, 1.192–1.378; *P* < .001). Notably, the *I*^2^ statistic showed significant heterogeneity among the included clinical studies (*I*^2^ = 58.3%, *P* = .019).

**Figure 3:**
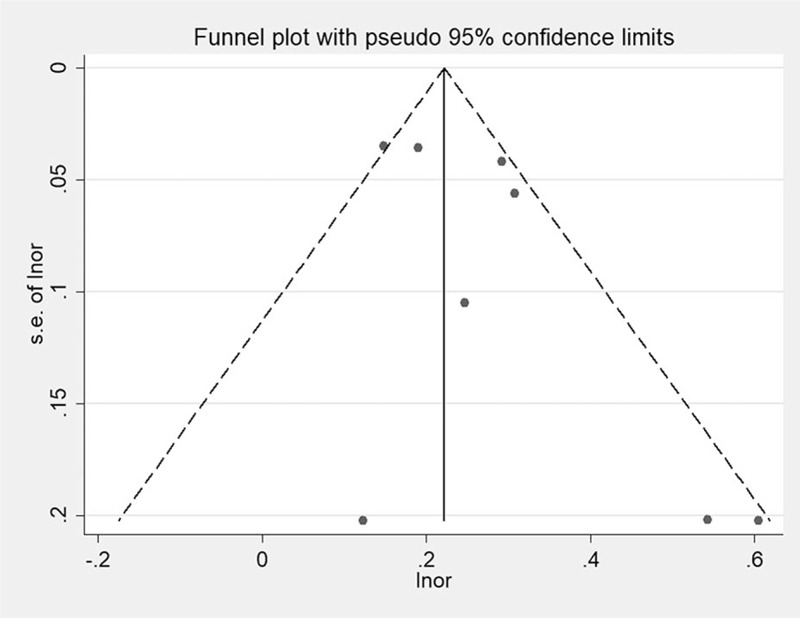
Funnel plots to test for publication bias.

The results of Begg's test (*P* = .458) and Egger's test (*P* = .152) indicated no significant evidence of publication bias (Fig. 3).

### 3.4 Subgroup analysis

The aforementioned results showed that *I*^2^ = 58.3% (*P* = .019) which indicated the significant heterogeneity among the included clinical studies. In order to figure out the parameters that may affect the stability and reliability of pooled analysis, the subgroup analysis was performed to investigate the potential factors causing the heterogeneity.

**Figure 4:**
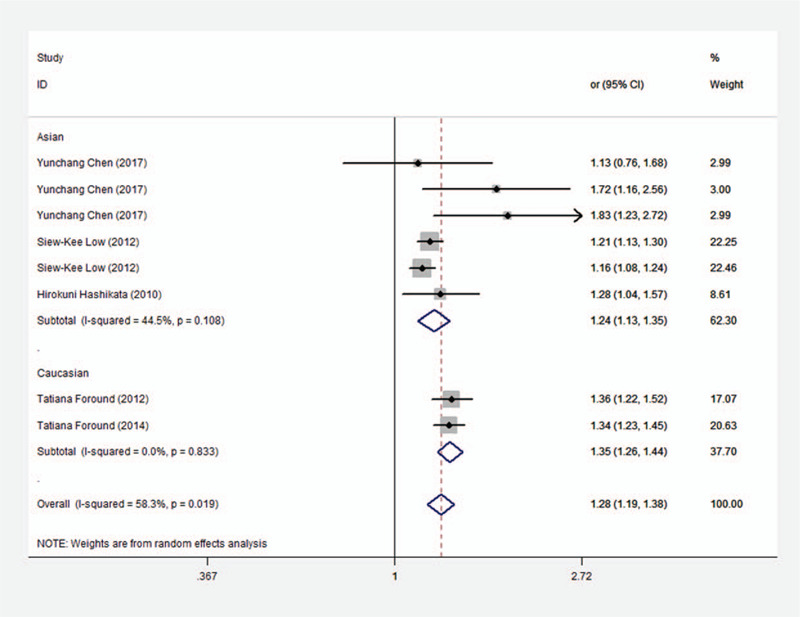
Subgroup analysis: stratified by Asian and Caucasian population.

As we know, several studies have proven the racial difference in diseases occurrence. Hence, we first speculated that race in each study may be an important factor influencing heterogeneity. As mentioned above, two studies investigated the association in Caucasian,^[9,16]^ others investigated the association between CDKN2BAS polymorphisms and intracranial aneurysm in Asian population.^[2,3,12]^ As shown in Figure 4, the results of the association were still stable in subgroups (Asian: OR, 1.24; 95% CI, 1.13–1.35; *P* < .001; Caucasian: OR, 1.35; 95% CI, 1.26–1.44; *P* < .001). Moreover, we also found that the heterogeneity shown no statistical difference in subgroups (Asian: *I*^2^ = 44.5%, *P* = .108; Caucasian: *I*^2^ = 0.0%, *P* = .833). Since the *I*^2^ value was significantly decreased compared to which in pooled analysis (*I*^2^ = 58.3%, *P* = .019). It indicated that the heterogeneity was significantly decreased after subgroup stratification. These outcomes indicated that race might be an important factor influencing heterogeneity.

**Figure 5:**
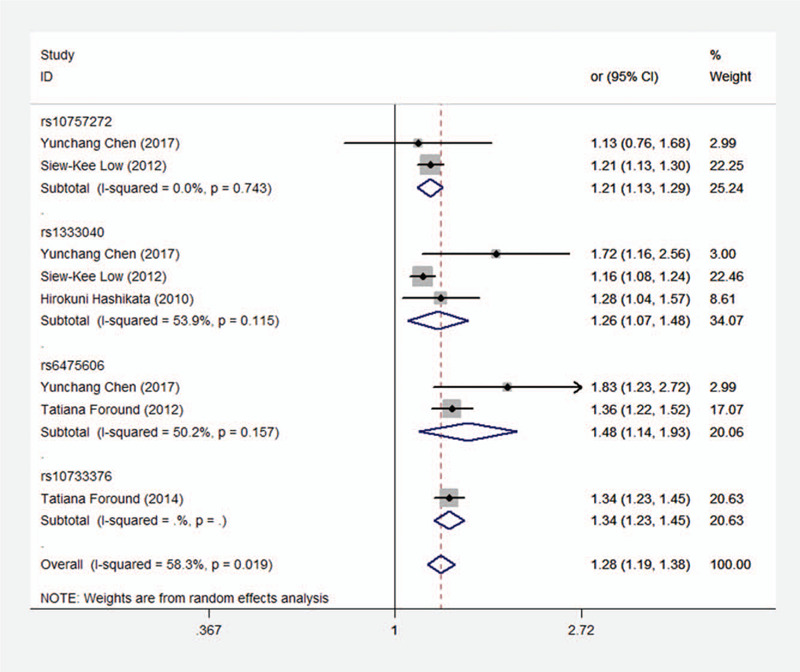
Subgroup analysis: stratified by rs10757272, rs1333040, rs6475606, and rs10733376.

In addition, because of the variant CDKN2BAS SNPs in the present research, studies were divided into four subgroups (rs10757272, rs1333040, rs6475606, and rs10733376). The results indicated that rs10757272 was strongly associated with intracranial aneurysm (Fig. 5, OR, 1.21; 95% CI, 1.13–1.29; *P* < .001). The *I*^2^ statistic also show no significant heterogeneity (*I*^2^ = 0.0%, *P* = .743). Likewise, similar results were shown in rs1333040 group (Fig. 5, OR, 1.26; 95% CI, 1.07–1.48; *P* = .005; *I*^2^ = 53.9%, *P* = .115) and rs6475606 group (Fig. 5, OR, 1.48; 95% CI, 1.14–1.93; *P* = .005; *I*^2^ = 50.2%, *P* = .157). In rs10733376 group, the *P* values and *I*^2^ values were not shown because of the insufficient quantity (Subjects ≤2). These results indicated that different CDKN2BAS SNPs might be another important factor influencing heterogeneity.

## 4 Discussion

As mentioned above, it is generally accepted that intracranial aneurysm is a disease associated to genetic alterations. SNP, defined as a substitution of a single nucleotide that occurs at a specific position in the genome, increases the complexity of the relationship between disease and genes. Fortunately, due to advances in technology, researchers can explore these complex connections at a deeper level. Several studies using GWAS have found chromosomal fragments that may be involved in the onset or rupture of intracranial aneurysms.

In this study, we aim to explore the CDKN2BAS SNPs (rs10757272, rs1333040, rs6475606, and rs10733376) genetic relationship with intracranial aneurysm, the results showed that rs10757272 was strongly associated with the disease. CDKN2BAS, a 3.8 kb long non-coding RNA (lncRNA) that located in chromosome 9p21,^[17]^ has been reported to be closely related to a range of diseases including a variety of cancers, diabetes, and aneurysm.^[3]^ Siew-Kee Low et al has found rs10757272 on CDKN2BAS at chromosome 9p21.3 to be significantly associated with intracranial aneurysm in the Japanese population.^[2]^ It is worth noting that rs10757272 was also reported to be associated with coronary artery disease (CAD) and platelet reactivity in the European population.^[18,19]^ In a study involved with the asymptomatic community-based Korean populations, researchers demonstrated that rs10757272 related to severe coronary artery calcification (CAC) based on each age and sex.^[20]^ This proves that it might be a common genetic risk factor in some cardio-cerebral-vascular diseases. However, due to the differences of genes in different populations, it is necessary to consider these heterogeneities in the analysis of the importance of these results. Though belonging to the Asian population same as Koreans and Japanese, no association of the variant rs10757272 with sporadic intracranial aneurysm in Chinese was shown in Yunchang Chen et al's study, the reason is probably due to genetic heterogeneity in different ethnic populations and may also be relevant in a bias caused by variation in the prevalence of a positive family history between populations.^[3]^

In addition to rs10757272, we found that rs1333040 and rs6475606 were also associated with intracranial aneurysms, which is consistent with previous experimental results, though the effect of them was less significant than rs10757272. In the study by Nakaoka et al, rs1333040 was identified as an independent predictor of intracranial aneurysms among other SNPs in the 9p21 region.^[21]^ Genetic variants of rs1333040 can also be an important risk factor in multiple intracranial and extracranial diseases. For example, Sturiale et al demonstrated an association between rs1333040 in the chromosomal 9p21 locus and sporadic brain arteriovenous malformations (BAVMs).^[22]^ This result was further confirmed by experiments conducted by Bendjilali et al. They found that these genetic found in BAVMs were similar to that in aneurysms, the reason may be attributed to the similar vascular pathology mechanisms between them. It is been also reported that rs1333040 affects the progression of coronary atherosclerosis and the probability of undergoing subsequent coronary artery revascularization after a first myocardial infarction whereas results of cardiovascular death was not affected.^[23]^ It is worth noting that rs6475606, another SNP we found to be statistically significant, has also been shown to be associated with perioperative myocardial injury after coronary artery bypass graft surgery. These results also give us inspiration that such genetic variants will not only be identified as risk factors for intracranial aneurysms, but also more widely involved in many other cardiovascular diseases.

There are still several limitations in the present meta-analysis. First, the whole analysis was based on the study level instead of individual level. Secondly, the quantity of included studies was not sufficient which may lower the credibility of results. In addition, the present study demonstrated that the variant CDKN2BAS SNPs and race were likely to be the potential sources of heterogeneity. However, the meta-regression was unable to be conducted because of insufficient data. That may cause the omission of other potential factors. Moreover, PCR approaches, study designs, sample sizes, etc varied a lot in the present study, these were also the inevitable flaws of meta-analysis. Hence, the conclusion of the present study should be interpreted and adopted with caution.

## Author contributions

**Conceptualization:** Ting Yu, Hailong Jiang, Ning Wang.

**Data curation:** Ting Yu, Hailong Jiang, Yunren Fan, Yunfeng Xu.

**Formal analysis:** Hailong Jiang, Yunren Fan, Yunfeng Xu.

**Investigation:** Hailong Jiang, Ning Wang.

**Methodology:** Ting Yu, Hailong Jiang, Ning Wang.

**Supervision:** Ning Wang.

**Validation:** Yunren Fan.

**Visualization:** Yunfeng Xu, Ning Wang.

**Writing – review & editing:** Yunfeng Xu, Ning Wang.

**Writing – original draft:** Ning Wang.
